# Expression analysis of cyclin D, Ki-67, MCM3 and MCM2 in oral squamous cell carcinoma

**DOI:** 10.6026/973206300191405

**Published:** 2023-12-31

**Authors:** Dushyantsinh Vala, Tarang Mehta, Mahesh Arjun Gadakh, Mimansha Patel, Animesh Mondal, Bharti Gupta

**Affiliations:** 1 Department of Oral and Maxillofacial Pathology and Oral Microbiology, Government Dental College and Hospital, Jamnagar, India; 2 Department of Oral & Maxillofacial Pathology and Oral Microbiology, Narsinhbhai Patel Dental College & Hospital, Sankalchand Patel University, Gujarat, India; 3 Department of Oral Pathology & Microbiology, SMBT Institute of Dental Sciences & Research., Dhamangaon, Nashik, India; 4 Department of Oral Pathology and Microbiology, Triveni Dental College, Bodri, Bilaspur, Chhattisgarh, India; 5 Department of Oral and Maxillofacial Pathology, Dr. R. Ahmed Dental College & Hospital, Kolkata, West Bengal, India; 6 Department of Maxillofacial Surgery and Diagnostic Sciences, College of Dentistry, Jazan University, Jazan 45142, Saudi Arabia

**Keywords:** Cyclin D, mini-chromosome maintenance molecules, MCM3, MCM2 and Ki-67, oral squamous cell carcinoma

## Abstract

The expression analysis of cyclin D1, Ki-67, MCM3 and MCM2 in oral squamous cell carcinoma to identify biomarkers is of interest. 45 formalin-fixed paraffin embedded tissue blocks collected from archives of Department of Oral and Maxillofacial Pathology and Oral Microbiology, Government Dental College and Hospital, Jamnagar, India were subjected to a retrospective cross-sectional immuno-histo-chemistry examination. 30 blocks of OSCC with histological diagnosis have 15 tissue blocks of well-differentiated oral carcinoma and 15 tissue blocks of moderately-differentiated oral carcinoma. 15 specimens of normal oral mucosa (NM) were also examined for comparison. In each of the categories, the immuno-histo-chemistry expression of cyclin D1, MCM 3, MCM 2, and ki67 was studied. Data shows that cyclin D1, Ki-67, MCM3 and MCM2 effectively indicate cellular proliferation for consideration as potential biomarkers of oral squamous cell carcinoma.

## Background:

Prognosis and diagnosis of cancer is critical [1,2]. A survivability rate of as high as 80% is reported for oral squamous cell carcinoma (OSCC) cases detected at the initial phase (T1N0); however, at later stages (T3-T4), this percentage drops to roughly 20%-30% [3,4]. Research has confirmed that the development of genetic mutations and epigenetic anomalies in the functioning of genes associated with cell proliferation is the initial step towards oral carcinogenesis [5,6, 7,8,9]. Therefore, evaluating the behaviour of the tumour, the prognosis, and the survival rate of patients require an understanding of cell proliferation [10-11]. To identify and measure the cell proliferation in oral cancer, a variety of proliferation biomarkers were established. In fact, research done in OSCC has shown the highest correlation between cyclins and cancer development [12,13]. The G1 phase, which is the sole stage in which outside influences like growth regulators can affect the cell cycle, seems to be crucial for cyclin D1 among the various cyclins [14,15]. There have been reports of cyclin D1 up-regulation and amplification in oral, head and neck, nasopharyngeal and laryngeal cancers [16,17]. Similarly, a small number of studies showed that mini-chromosome maintenance (MCM) molecules can be utilised as proliferation biomarkers to assess tumour behaviour due to their activity in the initial phase of G1 [18,19]. The proliferative scale and the prognostic aspect of OSCC patient longevity may both be estimated using MCM2 protein [20, 21].

One key factor in tumour progression, the proliferative potential of neoplastic cells, is thought to be a significant prognostic predictor [14-16]. Like another polypeptides that make up the MCM intricate, the MCM3 protein is found at reduced intracellular concentrations in differentiated and quiescent cells. Traditional indicators of cell division, that include the protein Ki-67, are frequently employed in the evaluation of a range of malignancies in humans, including neoplasms of the breast and prostate, sarcomas and lymphomas [22- 23]. MCM3 protein expression persists for longer compared to Ki-67 and it offers more accurate and sensitive data for assessing the proliferative characteristics of cell populations [19- 21]. In addition, it has been noted that the level of expression of the Ki-67 protein provides unclear knowledge concerning malignant neo-plasia because it includes the entire percentage of cells in the cell cycle, irrespective of whether or not these cells will ultimately differentiate in the absence of any correlation to a malignant form [22- 23]. Furthermore, in apoptotic cells or in situations where DNA synthesis is inhibited, Ki-67 activity may also happen [24,25,26]. It has been shown that certain human tumours, including salivary gland tumours, thyroid papillary carcinoma and melanoma engage MCM proteins as indicators of cellular proliferation [12,18, 20]. MCM2 protein positivity is thought to be a more accurate indicator of tumour prognosis in OSCC compared to Ki-67 [23-24]. Therefore, it is of interest to document the expression of cyclin D, Ki-67, MCM3 and MCM2 in oral squamous cell carcinoma to glean biomarkers.

## Methods and Materials:

## Design of the study and selection of patient:

Forty five archival collected formalin-fixed paraffin embedded tissue blocks were subjected to a retrospective cross-sectional immunohistochemistry examination. Thirty blocks of OSCC with histological diagnosis were included in the study that included fifteen tissue blocks of well-differentiated oral carcinoma and fifteen tissue blocks of moderately-differentiated oral carcinoma. Fifteen specimens of normal oral mucosa (NM) were examined (Table 1). In each of the two categories, the immuno histo-chemistry expression of cyclin D1, MCM 3, MCM 2, and ki67 was examined.

## Immunohistochemistry:

The conventional IHC method was used to assess the expression of cyclin D1, MCM3 MCM2, and Ki-67 using the following antibodies (Table 2). The tonsils for cyclin D1, MCM3, MCM2, and ki-67 were included in the positive control sections, which received the same treatment as the test categories.

## Immunohistochemical analysis:

Positive staining was demonstrated by the existence of a brown-colored final product at the target antigen location. Nuclear staining varied in intensity across all cases. The staining intensity was examined to determine the degree of stain absorption. On each slide, ten randomly chosen fields were magnified at a magnification of x40. The following scale was used to rate the staining intensity of each section [11-12].

No stain = 0

Mild stain= 1

Moderate stain = 2

Intense stain= 3

After scanning the complete section of the epithelium, the area of coloured epithelial cells was measured in order to ascertain the expression profile and the amounts of protein accumulation in the epithelial layers [17].

0% = 0

25% = 1

25%-49% = 2

50%-74% = 3

75%-100% = 4

After the slides were viewed at x40 magnification using an Olympus CX21 light microscope, illustrative photomicrographs were made of each slide in five different hotspot areas in order to immunohistochemistry examination. Thirty blocks of OSCC with histological diagnosis were included in the study that included fifteen tissue blocks of well-differentiated oral carcinoma and fifteen tissue blocks of moderately-differentiated oral carcinoma. Fifteen specimens of normal oral mucosa (NM) were examined (Table 1). In each of the two categories, the immuno histo-chemistry expression of cyclin D1, MCM 3, MCM 2, and ki67 was examined.

calculate the labeling index (LI). After that, an image processing programme called ImageJ (http://imagej.nih.gov/ij/) was used to analyse the photomicrographs. The total number of tumour cells in each slide was computed until a minimum of 400 cells were attained, i.e addition of the denominators (x). The percentage of IHC positive tumour cells per hot spot (A) was also determined [8].

The following formula was used to compute LI [9].

LI % = Ax100/Nooftumourcells

## Statistical Analysis

Statistical analysis was performed on the collected data using SPSS Version 17.0 (SPSS, Inc., Chicago, IL, USA). After doing a Mann-Whitney U test and Kruskal-Wallis ANOVA, P &kt; 0.05 was deemed statistically significant.

## Results:

The intensity of staining in normal mucosa and OSCC for MCM 3 and MCM 2 was greater as compared to ki-67 and cyclin D1. When compared among MCM 2 and MCM 3, it was observed that intensity of staining was greater in MCM 3 while among ki-67 and cyclin D1, intensity of staining was greater in cyclin D1. The intensity of staining in cyclin D1, MCM 3, MCM 2 and Ki-67 in normal mucosa was 1.611±0.818, 2.211±0.738, 1.422±0.696 and 2.012±0.738 respectively. The intensity of staining in cyclin D1, MCM 3, MCM 2 and Ki-67 in OSCC was 2.561±0.998, 2.900±0.627, 2.349±0.887 and 2.600±0.505 respectively (Table 3 and Table 4).

The area of staining in normal mucosa and OSCC for MCM 3 and MCM 2 was greater as compared to ki-67 and cyclin D1. When compared among MCM 2 and MCM 3, it was observed that area of staining was greater in MCM 3 while among ki-67 and cyclin D1, area of statining was greater in cyclin D1. The area of staining in cyclin D1, MCM 3, MCM 2 and Ki-67 in normal mucosa was 1.621±0.818, 1.831±0.786, 1.490±0.616 and 1.600±0.564 respectively. The intensity of staining in cyclin D1, MCM 3, MCM 2 and Ki-67 in OSCC was 2.461±1.410, 3.361±1.181, 2.249±1.294 and 3.149±1.061 respectively. The findings were statistically significant (p=0.033) (Table 5 and Table 6).

The values of labelling index (LI) in normal mucosa and OSCC for MCM 3 and MCM 2 were greater as compared to ki-67 and cyclin D1. When compared among MCM 2 and MCM 3, it was observed that LI values was greater in MCM 3 while among ki-67 and cyclin D1, values of LI was greater in cyclin D1. The values of LI in cyclin D1, MCM 3,MCM 2 and Ki-67 in normal mucosa was 9.53± 4.109, 19.442± 2.882, 7.31± 2.017 and 17.220± 2.660 respectively. The values of LI in cyclin D1, MCM 3,MCM 2 and Ki-67 in OSCC was 22.159 ±6.547, 44.643± 12.794, 20.037±4.325 and 42.421 ± 10.578 respectively. The findings were statistically significant (p=0.011) (Table 7)

## Discussion:

More than ninety percent of malignant oral neoplasms are OSCCs, which are part of a wider grouping of tumours called HNSCCs. The International Agency for Research on Cancer reports that India has a prevalence rate of 12.6/100,000 cases of oral cancer [12,14]The elevated prevalence of OSCC in India has been linked to multiple etiological variables, including alcohol intake, tobacco chewing of tobaaco, smoking habits, and human papillomavirus infections. These elements may contribute to the development of oral cancer separately or in combination [15, 16]. As the tumour grows, the chances of survival decrease, hence it is imperative to detect it as soon as possible. For cases of oral squamous cell carcinoma (OSCC) found in the early stages, a survivability rate of up to eighty percent has been observed; however, at later stages, this percentage falls to about twenty percent to thirty percent [21-24]. Studies have shown that the first step towards the development of oral carcinogenesis is the emergence of genetic mutations and epigenetic abnormalities in the regulation of genes linked to cell proliferation [12-16]. Thus, an awareness of cell proliferation is necessary in order to assess the behaviour of the tumour, the prognosis, and the survival rate of patients [13-17]. Many proliferation markers were developed in order to detect and quantify the cell proliferation in oral cancer. In fact, studies conducted in OSCC have revealed the strongest link between cyclins, MCMs and the onset of cancer [16-21].

The purpose of the current investigation was to compare and analyse the immuno-expression of cyclin D, MCM3, MCM2 and Ki-67 in order to determine whether or not these proteins may be used as indicators of cellular proliferation in OSCC. The values of labeling index (LI) in normal mucosa and OSCC for MCM 3 and MCM 2 were greater as compared to ki-67 and cyclin D1. When compared among MCM 2 and MCM 3, it was observed that LI values was greater in MCM 3 while among ki-67 and cyclin D1, values of LI was greater in cyclin D1. The values of LI in cyclin D1, MCM 3,MCM 2 and Ki-67 in normal mucosa was 9.53± 4.109, 19.442± 2.882, 7.31± 2.017 and 17.220± 2.660 respectively. The values of LI in cyclin D1, MCM 3,MCM 2 and Ki-67 in OSCC was 22.159 ±6.547, 44.643± 12.794, 20.037±4.325 and 42.421 ± 10.578 respectively. The findings were statistically significant (p=0.011).

A previous study claim that MCM3 protein expression provides more sensitive and accurate information for evaluating the proliferative traits of cell populations than Ki-67 since it is expressed for a longer period of time [13- 23]. Furthermore, as noted by previous study, the expression level of the Ki-67 protein encompasses the total percentage of cells in the cell cycle, regardless of whether or not these cells will eventually differentiate into a malignant form, thus providing ambiguous information regarding malignant neoplasia [17- 23]. Moreover, Ki-67 activity can also occur in apoptotic cells or in conditions where DNA synthesis is suppressed [21-24].Research has demonstrated that MCM proteins function as markers of cellular proliferation in a number of human cancers, such as melanoma, thyroid papillary carcinoma, and salivary gland tumours [12-19]. Compared to Ki-67, MCM2 protein positivity is regarded to be a more reliable measure of the prognosis of the tumour in OSCC [10- 14].

The role of MCM3 in oral cancer is not known. The area of staining in normal mucosa and OSCC for MCM 3 and MCM 2 was greater as compared to ki-67 and cyclin D1. When compared among MCM 2 and MCM 3, it was observed that area of staining was greater in MCM 3 while among ki-67 and cyclin D1, area of staining was greater in cyclin D1. The area of staining in cyclin D1, MCM 3, MCM 2 and Ki-67 in normal mucosa was 1.621 ± 0.818, 1.831 ± 0.786, 1.490 ± 0.616 and 1.600 ± 0.564 respectively. The intensity of staining in cyclin D1, MCM 3, MCM 2 and Ki-67 in OSCC was 2.461±1.410, 3.361 ± 1.181, 2.249 ± 1.294 and 3.149 ± 1.061 respectively. The findings were statistically significant (p=0.033). Cyclin D1 has been reported to be amplified and overexpressed in head and neck, laryngeal, nasopharyngeal, and oral malignancies [10- 13]. Similarly, a small number of studies showed that because mini chromosome maintenance (MCM) molecules are active during the first phase of G1. They can be used as proliferation indicators to evaluate the behaviour of tumours [12-16].

MCM 2 protein can be used to determine both the proliferative scale and the prognostic component of OSCC patient longevity [14-17]. The proliferation potential of neoplastic cells is considered a critical element in the progression of tumours and a major prognostic predictor. The MCM3 protein is present in differentiated and quiescent cells at lower intracellular concentrations, much like the other polypeptides that comprise the MCM complex [13-17]. Conventional markers of cell division, such as the protein Ki-67, are widely used in the assessment of a variety of human cancers, such as prostate and breast neoplasms, sarcomas, and lymphomas [12-16]. In our study the intensity of staining in normal mucosa and OSCC for MCM 3 and MCM 2 was greater as compared to ki-67 and cyclin D1. When compared among MCM 2 and MCM 3, it was observed that intensity of staining was greater in MCM 3 while among ki-67 and cyclin D1, intensity of staining was greater in cyclin D1. The intensity of staining in cyclin D1, MCM 3, MCM 2 and Ki-67 in normal mucosa was 1.611±0.818, 2.211±0.738, 1.422±0.696 and 2.012±0.738 respectively. The intensity of staining in cyclin D1, MCM 3, MCM 2 and Ki-67 in OSCC was 2.561±0.998, 2.900±0.627, 2.349±0.887 and 2.600±0.505 respectively. Similar data is known [17-23]. The Ki-67 protein is one of the most studied markers of cellular proliferation. When cells enter G0, its expression abruptly ends. It starts in the S phase, grows steadily during the G2 phase, and peaks in the G1 phase [19-21]. The fraction of cells that express Ki-67 is frequently associated with the clinical progression of tumours, particularly breast and lung cancers [22-26].

Studies have demonstrated a correlation between tumours with elevated levels of Ki-67 expression and a decreased probability of survival and recurrence. In OSCC, Ki-67 has also been demonstrated to be a strong predictive indicator of survival and recurrence [21-24].A previous study claim that because the MCM protein complex is involved in cellular proliferation, modifications that lead to an increase in this helicase's activity are associated with the development of cancer [13-15]. Consequently, studies have demonstrated a negative prognosis for brain tumours, salivary gland tumours, thyroid carcinomas, melanoma, and twelve other types of malignancies when MCM3 expression is increased [15-19]. The mean LI of cyclin D1 and ki-67 was found to be lower in the current study than the mean LI of MCM3 and MCM2 in the study groups. This may be the result of the MCM2 and MCM3 proteins, which are produced in the nucleus of a cell from the initial G1 phase onward and which recognize both cycling as well as non-cycling cells having proliferative capacity across the cell cycle [15- 19]. Therefore, compared to cyclin D1 and ki 67, MCM2 and MCM 3 can be more useful biomarkers for cell proliferation in OSCC. To validate the current findings, more research with a bigger sample size is required.

## Conclusion:

Data shows that MCM3 and MCM 2 are potential biomarkers compared to cyclin D1 and Ki-67 in OSCC.

## Figures and Tables

**Table 1:** Distribution of study specimens

**S.No**	**Category**	**Type of specimens**	**No of specimens**
1	NM	Normal mucosa	15
2	OSCC	Oral squamous cell carcinoma	30 (15 moderately differentiated and 15 well differentiated

**Table 2:** Details of proliferative biomarkers and their antibodies

**Proliferative biomarker**	**Antibodies**	**Name of antibody**
Cyclin D1	anti-cyclin D1	rabbit monoclonal antibody - EP12
MCM3	Anti MCM3	Clone 101
MCM2	anti-MCM2	rabbit monoclonal antibody - EP40
Ki-67	Anti ki-67	Polyclonal

**Table 3:** Intra group comparison of cyclin D1, MCM 2, MCM 3 and Ki-67 intensity of staining between the study groups

	**Cyclin D1**		**MCM3**		**Ki 67**		**MCM2**	
	**Normal**	**OSCC**	**Normal**	**OSCC**	**Normal**	**OSCC**	**Normal**	**OSCC**
N	15	30	15	30	15	30	15	30
Mean	1.611	2.561	2.211	2.9	1.422	2.349	2.012	2.6
SD	0.818	0.998	0.738	0.627	0.696	0.887	0.738	0.505
Mean Rank	12.71	25.84	13.05	25.26	12.59	24.73	13.05	23.26
χ2	9.35		9.35		8.249		9.138	
P	0.017*		0.01*		0.015*		0.01*	

**Table 4:** Inter group comparison of cyclin D1, MCM 3, MCM 2 and Ki-67 intensity of staining between the study groups

	**Cyclin D1**	**MCM3**	**Ki 67**	**MCM 2**	**P value**
NM	1.611±0.818	2.211±0.738	1.422±0.696	2.012±0.738	0.079
OSCC	2.561±0.998	2.900±0.627	2.349±0.887	2.600±0.505	0.266

**Table 5:** Comparison of cyclin D1, MCM 2, MCM 3 and Ki-67 area of staining between the study groups

	**Cyclin D1**		**MCM3**		**Ki 67**		**MCM2**	
	**Normal**	**OSCC**	**Normal**	**OSCC**	**Normal**	**OSCC**	**Normal**	**OSCC**
N	15	30	15	30	15	30	15	30
Mean	1.621	2.461	1.831	3.361	1.49	2.249	1.6	3.149
SD	0.818	1.41	0.786	1.181	0.616	1.294	0.564	1.061
Mean Rank	16.61	24.21	12.66	27.34	15.49	23.02	10.44	26.23
χ2	4.392		13.123		4.281		11.901	
P	0.118		0.002		0.116		0.001	

**Table 6:** Inter group comparison of cyclin D1, MCM 2,MCM 3 and Ki-67 intensity of staining between the study groups

	**Cyclin D1**	**MCM3**	**Ki 67**	**MCM 2**	**P value**
NM	1.621±0.818	1.831±0.786	1.490±0.616	1.600±0.564	0.636
OSCC	2.461±1.410	3.361±1.181	2.249±1.294	3.149±1.061	0.033

**Table 7:** Comparison of values of labeling index between cyclin D1, MCM 2, Ki 67 and MCM3 in normal mucosa, oral squamous cell carcinoma

	**Cyclin D1 LI**	**MCM3 LI**	**Ki 67 LI**	**MCM 2 LI**	**P value**
NM	9.53± 4.109	19.442± 2.882	7.31± 2.017	17.220± 2.660	0.001
OSCC	22.159 ±6.547	44.643± 12.794	20.037±4.325	42.421 ± 10.578	0.001
LI= Labeling index
